# An Evolving Understanding of Sense of Place in Social-Ecological Systems Research and the Barriers and Enablers to its Measurement

**DOI:** 10.1007/s00267-023-01882-1

**Published:** 2023-09-19

**Authors:** Joe Duggan, Christopher Cvitanovic, Ingrid van Putten

**Affiliations:** 1 Fenner School of Environment and Society, Australian National University, Canberra, ACT Australia; 2 Department of Pacific Affairs, Australian National University, Canberra, ACT Australia; 3 School of Business, University of New South Wales, Canberra, ACT Australia; 4 Oceans and Atmosphere, CSIRO, Hobart, TAS Australia; 5 Centre for Marine Socioecology, University of Tasmania, Hobart, TAS Australia

**Keywords:** Sense of Place, Social-ecological systems, Measurement, Practice

## Abstract

Social-ecological systems (SES) are changing more in the Anthropocene than ever before. With this also comes a change in Sense of Place (SoP), that is, the emotional bond that a person (or group of people) has with a place. This impacts how individuals and groups interact with a place (i.e., their behaviours) and respond to disturbance or change (i.e., their adaptive capacity). To understand how SoP is changing across space and time and to be able to compare this across social-ecological contexts, we must first take stock of how SoP is conceptualised so as to understand how to capture and measure the phenomena in a meaningful way (e.g., to inform policy). Based on in-depth qualitative interviews with leading SoP researchers (*n* = 17 from 8 countries) this paper aims to identify: (1) the current breadth of theoretical conceptualisations for SoP; (2) the methodologies that have been used to measure SoP in different contexts and settings; and (3) the barriers and (4) enablers to the use of different methodologies. Results show that there has been a change in how SoP has been conceptualised over time, whereby it was traditionally considered as something singular and limited, towards something much more dynamic. Results also show that diverse methods (both quantitative and qualitative) have been used to measure SoP, but the choice of method is often a result of resource constraints that limit research design. These findings suggest that broader collaboration among stakeholders and increased interdisciplinarity would undoubtedly lead to improved outcomes in our understanding of SoP, specifically how it is changing in response to anthropogenic pressures, and how the results can be integrated into policy and practice to support environment conservation and management. It is hoped these findings can help establish a community of practice around how we conceptualise SoP, and hence understand it, to create space for methodological integration and shared learnings as a field.

## Introduction

We live in a world where change is the new norm. The climate is changing, land use is changing, distribution of people, plants and animals are changing (Black et al. 2011; Foley et al. 2005; Parmesan 2006). This change is not just impacting physical elements of the environment, it is also impacting humanity (IPCC 2022). As social-ecological systems (SES) change, so do our people-place bonds and this presents a new and urgent need to explore and understand Sense of Place (SoP). The links between SES and SoP are well established in the literature and are layered and multidirectional (Duggan et al. 2023; Masterson et al. 2017a, 2019). That is, SoP can be an active driver of social-ecological processes and an outcome of them. For example, a change in a SES can impact an individuals’ SoP by impacting how they interact with, travel across, or emotionally connect to that place (Cunsolo Willox et al. 2012). In turn, an individuals’ SoP can impact how they interact with a SES which can, in some cases, lead to pro environmental behaviour (Alonso-Vazquez et al. 2019), or increased resilience against environmental change (Faulkner et al. 2018).

The concept of SoP has its origins in the 1970s, originally developed by phenomenological researchers, particularly the work of Tuan (1974, 1977). Broadly speaking SoP relates to the emotional bond that people have with a place (van Putten et al. 2018). It is an overarching term that encompasses other related concepts such as place attachment (*sensu* Masterson et al. 2017a, b) and place meaning (*sensu* Raymond et al. 2017). Early conceptualisations of SoP inferred a desired end point (Relph 1976), that is the more time you spend experiencing a given area, the more connected you are to it, with the end goal being stability—a way to give ‘moorings’ to our identities (Lewicka 2011; Raymond et al. 2021).

These initial conceptualisations and applications of SoP began to draw criticism in the 1980s as being too bounded and parochial (see Antonsich 2011). There were calls, therefore, for SoP to shift towards something that was more outward-looking and relational (Massey 1991). At the same time a new view of SoP was emerging from the realm of social psychology, where SoP was posited to be something more dynamic, flexible, and changing (Di Masso et al. 2019). It could evolve over time and was subject to cause-and-effect relationships (Masterson et al. 2017a) where changes to people and/or place could alter SoP (Raymond et al. 2017).

Since this time definitions of SoP have continued to evolve, with Raymond et al. (2021) recently calling for a rethink of our existing concepts of SoP given the drastic rate at which the places around us, and how we experience them, are changing. Suggesting a move towards pluralising the phenomena: *“… an epistemic attitude that is sensitive to the multiple knowledge-production strategies and conceptualisations that try to account for how senses of place are forged […] Pluralising sense of place offers scholars a powerful lens for translating these large and complex global challenges into multiple consequences relevant to local communities”*. This evolving definition of SoP does not have to exclude the more fixed conceptualisation, in fact current research acknowledges the tension between fixity and fluidity/flow in SoP (Devine-Wright et al. 2020) and largely speaking phenomenological and social psychology researchers acknowledge the validity of either approach (see Stedman 2016). Indeed, the diversity of varying conceptualisations can, and should, be considered as complimentary (Masterson et al. 2017a).

This thread of evolving conceptualisations highlights some of the challenges the field is facing. Specifically, evolving and emergent understandings of SoP present challenges to how we measure and build policies that account for it in SES management. A recent review of the literature on SoP highlighted that while significant steps are being taken in SoP measurement within SES, there remain key gaps (Duggan et al. 2023). They found that a range of approaches have been adopted in recent times, from surveys (Dor et al. 2014; Marshall et al. 2012) and interviews (Asfaw et al. 2019; Masterson et al. 2017b) through to Public Participation Information Systems (PPGIS) (Brown and Reed 2012) and photo-elicitation (Pérez-Ramírez et al. 2019), but to date there has been no work specifically exploring why these approaches are being used in any given context, nor how to compare results across contexts. The authors call for the development of a framework that would allow for consistency and comparison of results between studies, creating a community of practice or a group with shared interests that may interact and learn from one another (Vincent et al. 2018; Wenger 1988). This term was borne out of the fields of anthropology and education but has been applied across a range of contexts in recent times (Wenger-Trayner and Wenger-Trayner 2015).

We believe the first step in this journey is to understand what methodologies researchers are using to measure SoP and to understand the barriers and enablers for their use. In light of this, this paper aims to capture the learnings of leading SoP researchers to identify (1) the breadth of conceptualisations for SoP, (2) the methodologies that have been used to measure SoP, and (3) the barriers and (4) enablers to their use. To address these questions, we use in-depth qualitative interviews of academic researchers with experience in SoP research. Ultimately it is hoped that this work can help establish a community of practice around how we conceptualise SoP, and hence understand it, to create space for methodological integration and shared learnings as a field. It is hoped that this can then aid in identifying future priority research areas, inform funding allocation to the space, allow for cross-context comparisons, and enhance utility to policy for the management of SES.

It is also hoped that a deeper understanding of SoP can aid and inform SES research more broadly. Firstly, incorporating SoP into SES research allows us to include a level of ordered flexibility and subjectivity into the research. That is, by considering SoP within SES we may be able to more accurately comprehend the ‘social’ side of a system (see Stedman 2016 for a full exploration). Secondly the inclusion of SoP as a variable that influences individuals and communities’ responses to changes in SES can aid in understanding the overall resilience of a system (Eakin et al. 2016; Masterson et al. 2019). That is, by understanding SoP we may be able to more actively and effectively support communities and individuals to process and respond to environmental change (Eakin et al. 2019).

## Methods

### Methodological Approach

To address the aims of this study we opted for a qualitative research approach. Qualitative research approaches were considered optimal as they allow the research team to elucidate in-depth responses from participants, drawing on their deep expertise and experiential knowledge of working with SoP (Bryman 2016). Further, qualitive research approaches are more appropriate for capturing a plurality of framings (which, based on evolving definitions of SoP is clearly important) than is typically allowed by more quantitative research approaches. For example, qualitative methods allow interviewees to use their own words and can allow for ‘unexpected’ themes to emerge (Hay 2000).

### Participant Selection

Participants were initially identified from the pool of authors that contributed to the 62 papers identified as part of a recent systematic review of SoP measurement within SES (Duggan et al. 2023). Authors that appeared on two or more papers identified through that review were selected and then their personal research pages (i.e., formal institutional webpages, google scholar accounts, etc.) were reviewed to ensure they were still active members of the SoP research community. Authors that identified SoP or a derivative as a research focus and/or who had published a SoP paper in recent years were confirmed as potential participants. This process identified five individuals.

All potential participants were contacted via email and invited to participate in this study, with all agreeing to take part. To ensure comprehensive coverage of our participant group we also utilised snowball sampling (Noy 2008), whereby following each interview we asked the participant if they could recommend other researchers who, in their perspectives, had SoP as a focal research topic. This process yielded an additional ten experts who were invited to participate, seven of which agreed. Finally, lead authors from a recently published and seminal book about SoP were contacted (Raymond et al. 2021) and snowball sampling was adopted once more to ensure comprehensiveness of our sample size. This resulted in a further five experts.

We note that there is no universally accepted ‘best practice’ sample size when undertaking qualitative research. Evidence, however, suggests that theoretical saturation for qualitative interviews is commonly attained after 12 interviews, while meta-themes are regularly apparent with fewer (Guest et al. 2006). In this study we continued interviews until new data failed to provide new insights (Bryman 2006), that is, we reached theoretical saturation of ideas following the process described in Cvitanovic et al. (2016) at 17 interviews.

### Data Collection

An interview protocol was drafted to guide the interviews and ensure consistency in the questions that were asked of the participants (full interview protocol can be found at Appendix I). This was done via a discussion among the first two authors who drew out lines of questioning to address the aims of this study. The third author, who was also identified as a suitable participant for inclusion in the study based on the criteria outlined previously, was the first participant interviewed (i.e., pilot participant) and following the interview provided feedback on the interview guide to help refine and improve its clarity and purpose. Where unexpected themes emerged throughout the interview process, the guide was modified (following consultation between the first two authors).

Interviews were conducted over zoom and ranged in length from 30–75 min. All interviews were conducted by the first author. Detailed notes were taken in real-time throughout and the interviews were also recorded (with participant consent, and in line with ethical approval—see below) and professionally transcribed to ensure their accuracy for analysis purposes.

### Data Analysis

To allow themes to emerge from the data an inductive coding approach based on grounded theory analysis was adopted (Glaser and Strauss 1967). To do so, interview transcripts were imported into the software program Nvivo 12. To ensure accuracy of coding and account for intra-coder variability, initially two transcripts were coded by all three authors independently. The authors then came together and results were compared and discussed. A high level of agreement in the emergent themes was evident and any small differences were discussed before a resolution was agreed on by all authors—to guide future coding. Following this clarification process, all interviews were coded by the lead author.

Throughout the grounded theory analysis ‘in vivo’ codes were identified, that is, research participants’ direct statements (Charmaz 2008). This allowed for participants perspectives to emerge naturally without the constraints of deductive coding approaches that often set pre-defined ‘themes’. Following this, a second round of coding was conducted (by two authors, JD and CC) whereby the in vivo codes were grouped thematically to identify broad themes. To ensure validity new themes were continually compared against the raw data following previous studies (Blythe and Cvitanovic 2020; Cvitanovic et al. 2021; Kelly et al. 2019; Norström et al. 2020).

Throughout the coding process, the number of sources for each code was recorded (i.e., the number of interviews each code was recorded in). Given that frequency (i.e., the number of times a theme was coded across all interview transcripts) is not indicative of the importance that participants attribute to the specific theme it was not recorded (Cvitanovic et al. 2016, 2021). When coding for methods used by participants, related or overlapping methods were coded separately (i.e., if a participants indicated they used PPGIS as a methodology and part of this process involved adopting a survey, both ‘PPGIS’ and ‘survey’ were coded).

## Results

Analysis of the 17 interview transcripts revealed 18 themes that were mapped against the four research aims. The emergent themes against each aim are presented in Table 1 as an analysis hierarchy. It is important to note, however, that this does not indicate the level of importance that participants inscribed to each theme, only the number of participants that discussed the theme. Specific themes are detailed in the following subsections.

**Table 1:** Analysis hierarchy

Aim	Theme	No. participants that mentioned theme
Definitions and conceptualisations		17
	SoP as a broad concept	12
	SoP has tangible and intangible elements	10
	SoP is context specific and dynamic	10
	The contrast between individual and collective SoP	5
	SoP as a boundary object	4
Methodologies		17
	Qualitative	16
	Quantitative	11
	Modelling	1
Barriers to measurement		15
	Practical	10
	Research Pipeline	7
	Institutional	6
	Methodological	6
	Definitions	4
	Context and understanding	3
Enablers for measurement		12
	Collaboration and networks	6
	Research design	5
	Methodological advancements	4
	Changes to funding	2

Emergent themes and the number of study participants that mentioned each

### Conceptualisations of SoP

**Fig. 1:**
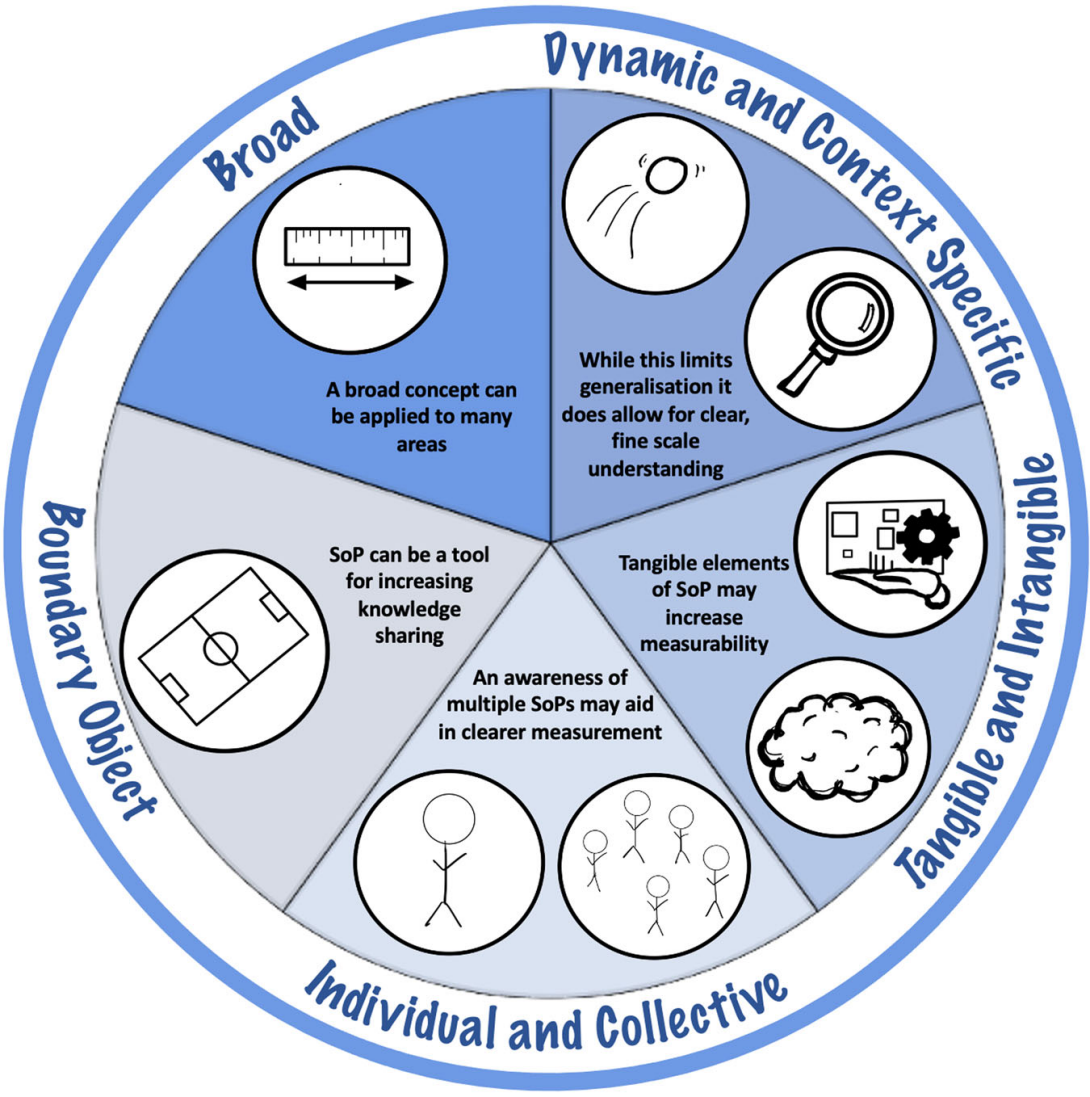
SoP has many overlapping and oftentimes contrasting conceptualisations. While at first appearing challenging, if approached in a considered way, it ultimately makes SoP a valuable and eminently useable phenomena

The first aim of this study sought to understand the diversity of ways by which SoP is conceptualised by experts working in the field. Every participant articulated a conceptualisation of SoP, which were coded into five overarching themes (see Fig. 1): (1) SoP as a broad concept, (2) SoP as context specific and dynamic, (3) SoP as consisting of tangible and intangible elements, (4) the individual and collective nature of SoP and finally (5) SoP as a boundary object.

Participants noted that there is no singular definition, but rather described SoP as a broad and overarching phenomenon (*n* = 12). In doing so, they recognised this as both as a benefit and a drawback for research in the field. For example, as one participant stated: *“I acknowledge that there are so many different understandings of senses of place […] this is very challenging, especially for young researchers to somehow find your way through that and find your own understanding.” (ID 12)*. In contrast, other participants considered this broadness as a strength, for example: *“…this concept is very broad enough and inclusive enough that you can really work with a lot of different ideas about what a place is in a very nice way…” (ID 15)*.

The second most commonly discussed conceptualisation was that SoP was dynamic, and dependent on context (*n* = 10). The dynamic nature of the phenomenon was often framed as a tension between fixity and fluidity, while the context specific nature of SoP was cited as a challenge for researchers: *“*[SoP has] *multiple layers of meaning […]some of which are subject to change, others are more fixed […] we are constantly navigating this fixity and fluidity in our connections to place over time.”* (ID 3). Another participant built on this by saying: *“…you need to understand* [SoP] *within a specific context because it can also be something different in different context. And I think that’s why people or scholars have failed throughout so many decades to really pinpoint what sense of place is.” (ID 16)*.

Participants of our study also highlighted that conceptualisations of SoP had features that were both tangible and intangible (*n* = 10), *“…it seems like the hidden dimensions of sense of place is actually what it is all about. The physical attributes just one aspect […] so my understanding of, besides that’s a very complicated multidimensional concept, is the fact that there’s tangible and intangible aspects around sense of place” (ID 16)*.

A less common theme when conceptualising SoP was the fact it can be experienced by both the individual and the collective (*n* = 5) and that these things can at times be at odds, *“…I mean, I talk a lot about feeling, it’s that personal feeling, but there’s also that affect, the kind of collective* [SoP] *(ID 14)”*. One researcher extended this to acknowledge a ‘dominating’ SoP. *“You can, of course, talk about a dominating sense of place that comes out in policy papers[…] in practice I would say, there is always those senses of place that comes to as many people you have around the table basically […] that have built a relationship to this specific locale. (ID 15)”*.

Finally, four participants conceptualised SoP as a boundary object. *“…and I think that’s where place becomes, I’m going to say powerful, in terms of bringing in a community together and sort of certifying what their meanings are that they care about.” (ID 10)*, and *“*[SoP allows] *you to kind of co-produce knowledge with them* [study participants] *if you involve them at the very early stage of your research, which it would increase your likelihood of success in what you’re doing.” (ID 2)*.

### Methodologies

The second aim of this study sought to identify the methodologies participants had used to measure SoP. It is worth noting at this point that on many occasions the term ‘measure’ caused confusion or contention with participants and needed to be clarified as being more than just quantitative methods. As outlined above, for our purposes to measure also included describing, understanding and articulating. *“I’m saying ‘measure’ because I doubt whether you can ever really measure it. But that’s my qualitative brain saying that. Some people believe you can measure it. I think you can measure aspects of it.” (ID 16)*.

Qualitative approaches were most commonly cited (*n* = 16), then quantitative (*n* = 11) and these were often combined within single studies. One researcher had only used modelling to investigate SoP.

Common qualitative methods like interviews, focus groups and workshops were most referenced (*n* = 12). Sensory methods were also commonly cited (*n* = 9), these were largely visual approaches such as photo-elicitation, drawing and model making but one researcher did note a shift towards soundscapes, *“the great thing about soundscapes is that it starts to address one of the big knowledge gaps in this scholarship, which is how do you empirically assess the temporal variable in one sense of the place? Because we can start to put sound monitors in the landscape for long periods of time, and then also ask people at given points in time how they relate to these sounds.” (ID 3)*.

Within qualitative methodologies, phenomenological approaches such as oral histories and firsthand description were referenced (*n* = 5). Participatory mapping was less common but was a feature of some participants approaches (*n* = 4), often used alongside other approaches, *“…we ask them, for instance, to map maybe their last visit to nature and then we have multiple questions, of course, relating to that. And one of the questions here is what does this place to mean?” (ID 15)*.

The most referenced quantitative approach was the use of surveys (*n* = 10). Again, these were often used in combination with qualitative approaches. One researcher spoke about starting with qualitative research to create a quantitative scale while another referenced the value of surveys for mapping *“…through deep interviews, through focus groups, we’ve developed a place meaning scale. This sounds weird because usually place meaning is qualitative or interpretive research[…] we actually developed through analysis, reanalysis, a quantitative scale” (ID 10)*. Interestingly, one participant had begun to explore SoP using theoretical modelling and attempting to incorporate SoP into ecological and socio-ecological models.

### Barriers to Measurement

The third aim of this study sought to explore the various barriers participants had experience when seeking to SoP. The majority of participants (*n* = 15) articulated a range of barriers. These responses could be grouped into six clear themes: practical barriers, research pipeline barriers, institutional barriers, methodological barriers, issues with definitions and challenges with context and understanding.

Practical barriers were the most commonly cited (*n* = 10) and included funding, time and ethical barriers. *“The first - the most important reason that we went for that approach is because we had no money to do any empirical analysis. So, the aim was to actually go to those places and explore those things empirically. But there was no money to do that.” (ID5)*. “*There’s the regular ones like, time constraints, resources, [researchers are] usually not expected to take three, four years to do one study and write that up.” (ID 6)*. Ethical barriers centred around questions of social justice and accountability for the researcher—for example, questioning whether seeing poor living conditions during a study meant that they needed to act against the situation.

Research pipeline barriers (*n* = 7) that is, barriers that impact the flow of research from idea to publication, were often articulated as a lack of expertise in a research team or a lack of professional experience for the researchers themselves. *“I think it’s hard to find people who can train you to do these methods well. Like I had access to some scholars… but otherwise you have to read up yourself, and […] I think there’s a lot of practical things in this type of research that requires more than just readings and previous studies.” (ID 6)*.

Institutional barriers (*n* = 6) also came back to money and ethics, but this time in the form of funding body priorities and ethics approval processes. *“…first, we wanted to do these workshops also for these environmental stewardship actions, but that was not something they would fund because this is not fundamental research. This is way too applied. I think it’s such a pity because it somehow, I mean, it connects.” (ID 12). “And there’s an ethical dimension to this too, that when you return to the same subject multiple times, over multiple years, the ethics […] increases, in terms of seeking approvals.” (ID 3)*. Other institutional barriers included the publish or perish culture, a narrow view of what constitutes knowledge and knowledge generation and a lack of support for transdisciplinary research.

Methodological barriers were mentioned by a number of participants (*n* = 6). These were linked to the context specific and dynamic nature of SoP and included a lack of appropriate measurement tools, challenges with data interpretation and issues with identifying comparable places. Issues of definition were cited as barriers on a number of occasions (*n* = 4), respondents felt that the broad and often intangible nature of SoP made it hard to capture and categorise. *“…the most difficult aspect of sense of place, particularly for people who are hoping to measure it, is that it is largely ineffable, it’s largely intangible, it’s largely invisible.” (ID 11)*.

Related to this, the final barrier was framed as an issue of connection and understanding (*n* = 3). This included the fact that SoP can mean different things to different groups or cultures *“I think there’s also challenges for our cross-cultural variations, in how do you engage with these concepts in a standardised way in different cultures, which have different understandings of these concepts?” (ID 3)*. Within this, some researchers felt an experience of a place or a strong connection to a particular SoP was crucial to be able to study it. *“I feel very hesitant to write papers based on just quantitative data, for example. I often feel like I […] want to go out and talk to people about what my tentative understanding of a situation is. Like, does this even make sense to you, does it fit with your world view, and so on. So, if I can’t sit down and just have a regular conversation with someone, that makes me very hesitant to go and write papers about them or the case.”* (ID 6).

### Enablers and Overcoming Barriers

The final aim of this study was to identify the enablers that improved participants ability to measure SoP. Fewer participants highlighted enablers compared to those that mentioned barriers (*n* = 12 vs. *n* = 15). Of the responses, four themes were evident: collaborations and networks, research design, methodological advancements and changes to funding.

Multiple researchers indicated that collaboration and building networks was an effective way to overcome barriers to measuring SoP (*n* = 6). These collaborations were seen as important across disciplines and across regions. *“I’m very much into interdisciplinary and interdisciplinary of not only of research, but also of education. So that would’ve been really useful to open my mind or my perspective in knowing, like, I don’t need to apply it necessarily, but I know that it exists, it works like that, and I am familiar with it, and it’s not just me trying to dig into something completely different that I haven’t even heard of before.” (ID 2)* and *“I think a lot of work could be done […] instead of funding better research from the north, is to fund more research that collaborates with local researchers…” (ID 6).*

Different research design considerations were flagged by multiple participants as ways of enabling effective measurement of SoP (*n* = 5). These included embracing mixed methods, considering scales in design and piloting research projects in the first instance. Related to this were the calls for methodological advancements (*n* = 4) such as further research into visual and immersive methods, performative methods along with technological developments. The final enabler mentioned by interviewees revolved around funding (*n* = 2). And included a call for more savviness from researchers when pitching for funds as well as a suggestion of greater involvement from researchers and practitioners in the creation of funding schemes.

## Discussion

SoP is a topic that has seen a drastic increase in scholarly attention in recent years (Duggan et al. 2023). With this attention comes a need to understand how best to account for and apply SoP in research and practice. This study builds on an earlier review (Duggan et al. 2023), that identified while significant steps are being taken in SoP research, there remain key gaps in understandings of both the conceptualisations and measurement of the phenomena across varied social-ecological contexts. We seek to address these gaps via in-depth interviews with SoP researchers to identify prominent conceptualisations of SoP as well as identifying common methodologies used to measure the phenomena and the barriers and enablers for their application. The following section explores these findings, situates them within the broader literature and discusses their implications for the field of research moving forward.

### Conceptualisations

As outlined in the introduction, SoP is a concept that has been explored, over time, from a range of distinct ideological standpoints and by numerous fields of research (Masterson et al. 2017a; Raymond et al. 2021; Stedman 2016; Tuan 1974, 1977). As a result, divergent conceptualisations of the phenomena have emerged. This is reflected by participants of this study, with results showing that there is no universally agreed single definition of SoP, but rather that it is a broad concept understood, applied and thus measured differently in different settings and contexts.

While a broad concept does lead to divergent conceptualisations, it also allows for researchers from diverse professional backgrounds to explore the phenomena, creating a springboard for increased interdisciplinarity and transdisciplinarity. Interdisciplinary research, or research where different academic disciplines work together to integrate knowledge and methods (Kelly et al. 2019), is often touted as a key approach in addressing contemporary challenges (Okamura 2019), particularly in SES (Blythe and Cvitanovic 2020). While transdisciplinarity, or research where academic and non-academic collaborators work together to integrate knowledge and methods (Kelly et al. 2019; Polk et al. 2015) is seen as a key element of meaningful co-production (Chambers et al. 2021; Norström et al. 2020), aiding the translation of research to practice (Duggan et al. 2023; Plummer et al. 2022). While such collaborative approaches stand to have many benefits for the field, they also have a history of reduced funding success (Bromham et al. 2016), bring with them cross-cultural challenges (Duggan and Sokini 2021) and regularly require an increase in time and resources to ensure the creation of meaningful relationships between researchers and disciplines (Cvitanovic et al. 2019; Steelman et al. 2021). One element of SoP that could aid in collaborative research approaches is its functionality as a boundary object.

Conceptualising SoP as a boundary object, or a tool for knowledge sharing (Kanwal et al. 2019), was an unexpected result that emerged throughout the interviews. Participants recognised that the phenomena could be used to bring communities together to share meanings and may well aid coproduction. Fully understanding the driver for this requires further work, but it is possible that the tangible and intangible elements of SoP allow the phenomena to retain an element of concreteness at the same time as being open to alternate interpretations (MacGillivray and Franklin 2015; Star and Griesemer 1989). This then positions SoP as a tool for knowledge sharing, coproduction and facilitation and many other applications. Such collaborative approaches have been shown to lead to improved knowledge use at the science/policy interface, are starting to be explored within SES research (Andersson et al. 2021; Arnott et al. 2020) and could well be the key to increased collaborative research.

A range of other conceptualisations were raised by participants throughout the study, from the contrasting tangible and intangible nature of SoP, to the context specific and dynamic nature of the phenomena and the fact it can manifest as something individual, or collective. These varying conceptualisations do not necessarily need to be seen as negatives, indeed as above they could be seen as features to aid shared learning and collaboration. They could also be features that allow for deeper understanding. For example, the intangible elements of SoP may make the concept hard to articulate and communicate, but the tangible elements perhaps have the opposite effect, reminding us that the physical reality of a space isn’t irrelevant (Stedman 2003), and it is perhaps this physical element that grounds the phenomena in reality enough to allow it to be measured at all. While further research is required to fully unpack these features, such diverse, contrasting and at times overlapping conceptualisations clearly present challenges when seeking to measure such phenomena.

### Methodologies

Considering the broad nature of SoP, it is unsurprising that participants referenced a broad range of methodologies for its measurement. Qualitative approaches were the most commonly cited. A high representation of interviews aligns with reviews of the literature (Duggan et al. 2023) and is common in place-based studies (Shackleton et al. 2021).

Participants often pointed to the depth and nuance that qualitative approaches can bring. The value of qualitative approaches in understanding the human experience has been recognised across a range of fields, from SES research (Biggs et al. 2021), to medical education research (Cleland 2017). While qualitative approaches generally require more time and resources for a relatively small sample size, they offer up the possibility of a deeper, more nuanced understanding of a given context (Anderson 2010)—an important consideration given how context dependent SoP appears to be.

Quantitative approaches were used less often by participants of this study, with surveys being the most regular approach. Again, this aligns with the literature and is a common feature of place-based studies (Duggan et al. 2023; Shackleton et al. 2021). The lower occurrence of quantitative approaches could be due to the limited depth of information that such approaches offer (Steckler et al. 1992; Verhoef and Casebeer 1997) or the inherent challenges of incorporating quantitative research into public policy (Jerrim and De Vries 2017), but this should not dissuade researchers from adopting quantitative methods altogether. Quantitative methods can often render larger sample sizes, can be scaled across regions, and could well allow for the measurement of SoP across a diverse range of contexts in a single study (Steckler et al. 1992; Verhoef and Casebeer 1997). If SoP is incorporated into modelling, like one study aimed to do, then quantitative data can be more easily incorporated to ascertain system level impacts or changes.

Often quantitative approaches were mentioned as part of projects where they were used in conjunction with qualitative approaches so as to add both breadth and depth to data, this is a feature of approaches such as participatory mapping (Brown et al. 2017) or when using a qualitative approach as the first step to then develop a quantitative scale (Evans et al. 2022). Incorporating both qualitative and quantitative approaches allows researchers to both explore and explain phenomena (Creswell and Plano Clark 2011) and as Greene (2015) puts it: *“a mixed methods approach to social inquiry provides more than one lens and perspective on the phenomena being studied and so promises better understanding of these phenomena”*. Although this is not a silver bullet, and conducting such research requires a sound rationale and an awareness of the barriers that influence the creation of truly mixed methods research (Bryman 2006, 2016).

### Barriers and Enablers for Measurement

In total our analysis identified six key themes of responses from participants referencing barriers to measurement. These were: practical barriers, research pipeline barriers, institutional barriers, methodological barriers, issues with definition and issues of context and understanding. These barriers are not ground-breaking and are often features of any complex research. They have been recognised across many areas of social-ecological research, from conservation (Bennett et al. 2017; Duggan and Sokini 2021) to environmental management (Reed 2008) and natural resource management (Cvitanovic et al. 2015). Many researchers have dedicated time and space to addressing these common barriers (Blythe and Cvitanovic 2020; Cvitanovic et al. 2015; Hein et al. 2018), and in light of this we are seeking to focus now on the enablers for SoP measurement—recognising the power of positivity and bright spots in furthering environmental science (Cvitanovic and Hobday 2018).

While commenting on enablers for the measurement of SoP, participants often came back to the importance of collaboration and networks. This could be construed a number of ways, all of which stand to benefit the field of research. First, a strengthening of networks through increased interdisciplinary research allows for complex social-ecological challenges to be better addressed (Alexander et al. 2019; Prell et al. 2007) leading to an increase in research impact (Okamura 2019). Second increased collaboration in the form of participatory research may improve knowledge sharing between knowledge generators and end users (Bednarek et al. 2018; Cvitanovic and Hobday 2018), and lead to more meaningful outcomes in research (Cash and Moser 2000; Moser 2016). In undertaking increased collaboration and networking, researchers are opening themselves up to the possibility of including more diverse knowledge systems into their practices (Moon et al. 2019). Traditional knowledge systems are being increasingly (and rightfully) recognised for their value in supporting sustainability transitions for SES (Tengö et al. 2017), but there remain challenges to implementing truly interdisciplinary research (see Hein et al. 2018 for an overview), not least of all individual and institutional level change is required (Goring et al. 2014) and this is an area that needs to be investigated further.

While mentioned only twice, the point of changes to funding is one that warrants unpacking. Funding models within science have long been criticised for their strong focus on rewarding publication rates and citations over collaborative, slower, longer-term research (Horta and Santos 2016; Nicholas et al. 2017; Warren 2019). Recent work has shown that funders can be leverage points for change, particularly in SES research, by escalating funding requirements for collaboration (Arnott et al. 2020). Perhaps SoP scholarship can be a focal point for a changing funding structure whereby funder, researcher and policy maker are more interactive.

### Study Limitations and Future Research

In this study we have begun to develop a deeper understanding of SoP and its measurement. There are however a number of limitations that must be considered. First, it must be noted that this study only included English speaking researchers, largely hailing from high income countries. While the majority of SoP academic literature is coming out of these locations (Duggan et al. 2023) and this pattern matches other areas of social-ecological research (Amarante et al. 2021; Maas et al. 2021), there is an inherent risk of reinforcing biases and skewing our understanding of the phenomena towards a Eurocentric focus (Moon and Blackman 2014; Nuñez et al. 2019).

Second, while it was not actively excluded, this study did not explicitly look at unpacking the relevance, involvement or overlap between SoP and Indigenous ways of knowing and being, this is undoubtedly a crucial area for future research. Certainly, there is research that explores how place is conceptualised differently across different cultures (Marshall 2022; Wilson 2003), and there is evidence that western ontologies can struggle to articulate Indigenous place and space (Country et al. 2015). But we also know that within our changing world, first nations people are being disproportionately impacted (Ford 2012) and in many instances this is impacting their SoP (Cunsolo Willox et al. 2012). Further research is needed and it needs to be done with caution and consideration not to force a western conceptualisation of place onto participants.

Next, this study is the first of its kind and while it represents a valuable step towards understanding SoP, the context dependant nature of phenomena means future research must validate these findings through repeat sampling. Future research should also extend the exploration of enablers and barriers to include the application of SoP. Extensive work has been done in understanding the science-policy interface (Cvitanovic et al. 2021; Karcher et al. 2021; Kolkman et al. 2016) but to date no research has been conducted into how and why SoP is (or isn’t) included in policy considerations.

Throughout the study a range of possible methods were brought to light by participants. From sensory, to participatory and performative—many of these methods were not yet in use by researchers, but they were referred to with a sense of optimism. Future research should address these in detail applying each and exploring their applicability across contexts. It is possible that thorough exploration could then lead to the creation of a heuristic or framework for identifying which methods will allow for the comparison in different contexts.

Finally, an area that warrants further research is the usefulness of artificial intelligence (AI) for the measurement of SoP. The functionality of AI to review, consider and measure large datasets has significantly improved in recent times and has found applicability in a range of fields and approaches, from evidence synthesis (Berrang-Ford et al. 2021) to environmental science and engineering (Zhong et al. 2021) and medicine (Kermany et al. 2018). AI presents an opportunity to measure SoP dynamically in new and novel ways (Kang et al. 2019; Song et al. 2021), this approach is in its infancy, but is an area of great promise.

## Conclusion

This area of research is marked with a range of research approaches, that are based off different assumptions (Masterson et al. 2017a; Stedman 2016) leading to some SoP literature being criticised as being messy or working at cross purposes (Stedman and Beckley 2007; Williams and Patterson 2007). Recent work has taken huge steps in unifying the field and setting a clear research direction (Raymond et al. 2021), but more work is required particularly in the area of measuring SoP. By conducting in-depth interviews with leading SoP researchers, we have been able to map a change in how SoP is conceptualised, that is, moving towards something much more dynamic. We have also identified that diverse methods have been used and that this is often driven by resource constraints. Researchers face a range of barriers when seeking to measure SoP from practical challenges like time and money through to a lack of expertise and institutional barriers. Broader collaboration and interdisciplinarity would undoubtedly address some of these barriers while improved research design and methodological advancements will likely make the measurement SoP easier and more effective. This work is the first step in establishing a community of practice, or *“groups of people who share a concern or a passion for something they do and learn how to do it better as they interact regularly”* (Wenger-Trayner and Wenger-Trayner 2015) that can explore together how best to conceptualise SoP, and hence understand it. The next step is to create space for methodological integration and shared learnings as a field. This can then facilitate the identification of future priority research areas, inform funding allocation to the space, allow for comparisons across SES and ultimately enhance utility to policy.

## Data Availability

The datasets presented in this article are not readily available to protect the privacy and anonymity of research participants, and in accordance with human ethics approvals related to this research, data cannot be provided unless required by law. Requests to access the datasets should be directed to corresponding author.

## Appendix I

**Interview Protocol**

**Preamble**

Welcome and thanks, talk about myself and my research, explain why I want to interview them.

**What is your understanding of SoP?**

**What is your experience of SoP?**

Prompt: Can you give me an example of where sense of place has mattered to you

**Definition of SoP:**

The emotional bond that people have with a ‘place’

SoP ‘embeds all dimensions of peoples’ perceptions and interpretations of the environment, such as attachment, identity or symbolic meaning, and has the potential to link social and ecological issues’.

1. **Introductory questions**
  - Tell me about yourself
  - Tell me about your Sense of Place (SoP) Research
2. **Understanding the participants motivations and drivers for studying SoP**
  - Why do you think studying SoP is important?
  *Prompt: Why do you study SoP?*
3. **Understanding how they measure SoP and why**
  - What methods have you used for measuring SoP before?
  - Why did you use those methods? Are there any other methods you considered?
  - Of the methods you did use, what were the advantages and disadvantages?
  *Prompt: internal/external/personally/other*
4. **Opportunities for improvement**
  - In a perfect world (if resources, time, money were not a limiting factor) how would you chose to measure SoP?
  - What are the barriers that have stopped you from doing this?
    *Prompt: Can you give examples?*
  - Can you think of specific ways to overcome these barriers?
5. **Opportunities for impact**
  - What are the opportunities for turning research into impact?
  - What are the opportunities to apply research for positive outcomes?
  - Do you think SoP is important for policy?
  - What are the barriers in including SoP in policy considerations?
    *Prompt: internal/external/personally/other*.
  - What are the enablers in including SoP in policy considerations?
  *Prompt: internal/external/personally/other*
6. **Conclusion**
  - Now that we have completed the formal component of the interview, are there other important issues that were not covered by our questions, or other relevant insights that you would like to share based on you experience?
  - And finally, considering the topic of the study, and the questions I’ve just asked you, who are three other people that you think I should interview in this research?

*Thanks and next steps*

## Appendix II

**Participant Information Sheet**

**Project Title**

Improving the application of Sense of Place to resource management

**General Outline of the Project:**

- Purpose and methods: The aim of this project is to address gaps in the measurement and application of Sense of Place for resource management. This will include the exploration the barriers and enablers to SoP measurement as well as the motivations and drivers for studying SoP. To address these questions, this study will use in-depth qualitative interviews of academic researchers with experience in applied SoP research.
- Use of Data and Feedback: We will use the data to contribute to scholarly publications/conferences.
- Research team:
  - Mr Joe Duggan—Primary Investigator
  - Dr Christopher Cvitanovic—Co-investigator
  - Dr Ingrid van Putten—Co-investigator

**Participant Involvement:**

- Voluntary Participation & Withdrawal: we invite you to participate in this research given your experience as a researcher of Sense of Place. You do not have to be involved in this research unless you want to, and you can pull out of the research if you change your mind without telling us why. If you do withdraw, we will not use the information you have provided at any time prior to finalisation of the study.
- What does participation in the research entail? You have been invited to participate because of your experience as a researcher of Sense of Place. Your participation will include an interview of ~45–60 min over zoom at a time of your convenience. Should you give written consent (please see consent form attached to this email), interviews will be audio recorded and professionally transcribed to ensure their accuracy. As such you will not be required to review your interview transcript, but can request to do so if you would like.
- Risks: participation in this study should involve no risks beyond those of everyday living. During all activities, you are under no obligation to answer any questions with which you are not comfortable, or provide any information you would prefer to withhold. Even if you agree to participant, you are also free to withdraw from the study at any point up until the results are finalised and published without having to provide an explanation. There is no penalty for refusing to participate in this study, withdrawing your participation at any point up until the project is finalised, or not answering specific questions. If, for some reason, participation in this project does cause you distress, we encourage you to email the contacts named at the end of this form who will assist you with identifying a suitable support avenue (e.g. counselling).

- Benefits: we expect participation will be enjoyable and meaningful, and will provide opportunities for participants to contribute to how SoP can be measured and applied. Through the publication of the outcomes of the project in academic journals, we will share the lessons from the project with the wider research community. To this end, the final published work will be shared with participants following finalisation of the project. However, there is no other direct benefit to participants from participating in the project.

**Confidentiality:**

Confidentiality will be protected as far as the law allows. To ensure confidentially, only the research team (named above) will have access to the raw data, and all of your responses to the interview will be processed so that unauthorised users cannot access them, in accordance with the Australian Human Research Ethics National Statement. To this end, we will not include any personal information in reports or papers and will seek to ensure that unless specific permission is obtained, comments or findings are presented in a way that is not individually identifiable. Although steps will be taken to protect the identity of the participants, there is a risk that others may guess the source of information. Therefore, participants should avoid disclosing information that is confidential, defamatory or that could otherwise harm their interests.

**Privacy Notice:**

In collecting your personal information within this research, the ANU must comply with the Privacy Act 1988. The ANU Privacy Policy is available at https://policies.anu.edu.au/ppl/document/ANUP_010007 and it contains information about how a person can:

- Access or seek correction to their personal information;
- Complain about a breach of an Australian Privacy Principle by ANU, and how ANU will handle the complaint.

**Data Storage:**

- Where and how long: data (including notes and analysis materials) will be stored for a minimum of 5 years following publication on ANU secure servers. De-identified hard copy materials will be stored in a locked filing cabinet accessible only to the research team. After 5 years, all raw data will be destroyed (i.e. computer files completely deleted, hard copy notes shredded, etc.).
- Handling of data following the required storage period: following all use of the data (i.e. reporting on outcomes of the project and contributing to academic publications), data will be retained by the ANU on secure servers.

**Queries and Concerns:**

- Contact Details for More Information: Enquiries about the project can be directed to Mr Joe Duggan via Joe.Duggan@anu.edu.au or +61 447 143 433.

**Ethics Committee Clearance:**

The ethical aspects of this research have been approved by the ANU Human Research Ethics Committee (Protocol 2022/307). If you have any concerns or complaints about how this research has been conducted, please contact:

Ethics Manager The ANU Human Research Ethics Committee The Australian National University Telephone: +61 2 6125 3427 Email: Human.Ethics.Officer@anu.edu.au

**Thank you for considering to participate in this study.**
